# First results from post-COVID inpatient rehabilitation

**DOI:** 10.3389/fresc.2022.1093871

**Published:** 2023-01-23

**Authors:** Alexa Kupferschmitt, Eike Langheim, Haris Tüter, Franziska Etzrodt, Thomas H. Loew, Volker Köllner

**Affiliations:** ^1^ Department of Psychosomatic Medicine, University Hospital Regensburg, Regensburg, Germany; ^2^ Psychosomatic Rehabilitation Research Group, Department of Psychosomatic Medicine, Center for Internal Medicine and Dermatology Charité – Universitätsmedizin Berlin, Berlin, Germany; ^3^ Department of Psychosomatic Medicine, Rehabilitation Center Seehof, Federal German Pension Agency, Teltow, Germany; ^4^ Department of Cardiology, Rehabilitation Center Seehof, Federal German Pension Agency, Teltow, Germany

**Keywords:** post-COVID-Syndrome, inpatient rehabilitation, treatment effect, psychosomatic, psychocardiology

## Abstract

**Background:**

COVID-19 is associated with various symptoms and psychological involvement in the long term. In view of the multifactorial triggering and maintenance of the post-COVID syndrome, a multimodal therapy with somatomedical and psychotherapeutic content is expedient. This paper compares the psychological stress of post-COVID patients and their course in rehabilitation to psychosomatic and psychocardiological patients.

**Method:**

Observational study with control-groups and clinical, standardized examination: psychological testing (BDI-II, HELATH-49), 6-MWT as somatic parameter, two measurement points (admission, discharge). Sample characteristics, including work related parameters, the general symptom-load and the course of symptoms during rehabilitation are evaluated.

**Results:**

At admission in all measures post-COVID patients were significantly affected, but less pronounced than psychosomatic or psychocardiological patients (BDI-II post-COVID = 19.29 ± 9.03, BDI-II psychosomatic = 28.93 ± 12.66, BDI-II psychocardiology = 24.47 ± 10.02). During rehabilitation, in all complaint domains and sub-groups, symptom severity was significantly reduced (effect sizes ranging from *d* = .34 to *d* = 1.22). Medium positive effects were seen on self-efficacy (*d* = .69) and large effects on activity and participation (*d* = 1.06) in post-COVID patients. In the 6-MWT, the walking distance improved by an average of 76.43 ± 63.58 meters (*d* = 1.22). Not a single patient deteriorated in walking distance, which would have been a possible sign of post exercise malaise (PEM).

**Conclusion:**

Post-COVID patients have a slighter psychological burden as psychocardiological or psychosomatic patients. Although rehabilitation is not curative, post-COVID patients benefit significantly from the interventions and there were no signs of PEM.

## 1. Introduction

The WHO (2021) defines post-COVID as persistent symptoms associated with the recovered SARS-CoV-2 infection. Post-COVID impairs the daily functioning of the affected persons, symptoms are still present at least 12 weeks after recovery and cannot be explained by another disease (1). Common symptoms are fatigue, dyspnoea, palpitation, cognitive deficits, pain, olfactory and gustatory disturbances, sleep disorders or psychological complaints such as anxiety or depressive moods (2). The number of symptoms at the acute COVID-19 phase is associated with anxiety and depressive long-term post-COVID symptoms (3). Studies also show that distress related to COVID-19 may have substantial negative impact and increase the risk of developing mental disorders (4, 5). Since the symptoms can affect different organ systems, the treatment of post-COVID-syndrome requires the combined expertise of pulmonology, cardiology or neurology and psychosomatic medicine. Post-COVID-syndrome affects up to 10% of COVID-19 patients (6, 7). Even if only a part of these patients needs rehabilitation, this will lead to a large demand considering the number of cases (about 30 million COVID-19 patients in Germany until july 2022). Since the post-COVID-syndrome is still a new clinical picture, there are hardly any indicative measures for treatment and causal therapy. However, the multimodal concept of rehabilitation is suitable for symptomatic improvement (8, 9). In Germany, psychosomatic rehabilitation is an established care model with a capacity of about 150,000 treatment places/year (10). It is mostly tried to use these existing structures and to adapt the already well-evaluated treatment programs to the specific needs of post-COVID patients (8, 11). One adapted concept is the multimodal treatment concept (12), which is embedded in the already existing and evaluated structure of psychocardiology (13). The multimodal treatment concept for patients with post-COVID-syndrome is in line with the research agenda of post-COVID fatigue (14) and with the Stanford Hall consensus statement for post-COVID-19 rehabilitation (15). Psychocardiology is a well-established integrative treatment concept for patients with comorbid mental and cardiac disorders (16, 17). Like psychocardiological patients, post-COVID patients have pronounced physical symptoms, which interact with psychological factors. The somatic symptoms cause significant psychological suffering but cannot yet be treated in a causative way. In order to treat both somatic and psychological sufferings, the combination of a detailed medical examination and care, psychotherapy and a well-measured, comprehensive exercise program is the most promising option for therapy. In addition, this multidisciplinary rehabilitation setting makes it possible to compare the severity and limitations of post-COVID patients with those of psychosomatic patients and those with physical as well as psychological symptoms, and to examine the course of treatment. In this context, opinions should be mentioned which warn of post-exercise malaise in the context of physical training (18, 19). Normally physical training is a central part of rehabilitation and usually effectively used for the purpose of fitness and muscle strengthening (20–22). Post exercise malaise is a phenomenon which postulates a so called crash after physical or mental (over)exertion and a frightening deterioration of the state of health. On the contrary, studies speak against the harmful effect of exercise in the context of fatigue (23). Nevertheless it is particularly important to generate valid data on the effectiveness and safety of rehabilitative programs. In order to contribute to the knowledge of rehabilitation options for post-COVID syndrome, this study addresses the following research questions:

1. Do post-COVID patients differ from psychosomatic patients or patients with heart disease and psychological comorbidity in their psychological symptom burden?
2. Do post-COVID patients benefit from rehabilitation in the same way as other patients (psychosomatic medicine, psychocardiology)?
3. Is there any evidence of worsening of physical health due to over-exertion (in the sense of Post-Exercise Malaise) as a result of rehabilitation?

## 2. Materials and methods

### 2.1. Sample and procedure

The observational study is based on clinical, standardised examination, including psychological testing (BDI-II, HELATH-49) and a physical parameter (6MWT), at two measurement points: admission and discharge. The study took place between September 2021 and End of March 2022, in the newly established post-COVID treatment concept (10), embedded in the psychocardiology department, with the advantage of equivalent psychosomatic and internistic care in a German rehabilitation clinic. The multi-modal rehabilitation concept included individual and group psychotherapy (CBT), individualised aerobic exercise training, body awareness training, breathing therapy, relaxation techniques and cognitive training as well as social counselling as core elements. All consecutively admitted rehabilitation patients who started rehabilitation were included. In case of Post-COVID, the previous course of infection was irrelevant for the inclusion in the study (e.g., mild symptoms to severe course with hospitalisation). Data were obtained as part of routine clinical diagnostics. Clinical diagnoses were based on ICD-10 as it is currently used in the billing of health insurance services in Germany. The ICD-10 diagnoses were made within the first two weeks of admission by the treating psychological psychotherapist or medical psychotherapist according to AMDP criteria, always reviewed by an experienced specialist (4-eyes principle). The diagnostic process was carried out by expert judgement based on psychopathological expertise, using observational information (e.g., formal thought processing, modulation of mood) and the the overal symptom pattern (24). Patients were asked for written consent on the use of clinical data for research purposes and informed about their rights to refuse data processing without indication of reasons or disadvantages to their treatment. Informed consent was given to the retrospective, anonymised data analysis upon admission to the clinic. In its statement of 19. Mai 2022, the Ethics Committee of the Brandenburg State Medical Association had no objections to this procedure.

**Eligibility criteria Post-COVID subsample:**

• SARS-CoV-2-infection and following post-COVID syndrome:
• Complaints that are present more than 12 weeks after the onset of SARS-CoV-2 infection and cannot be explained otherwise.
• As a consequence of the post-COVID syndrome, at the time of the start of rehabilitation, the presence of functional limitations that may threaten the ability to work.

**Eligibility criteria Psychosomatic subsample:**

• There is at least on diagnosis of any mental disorder, certified by the treating physicians, e.g. panic disorder, heart-related fears, depression.

**Eligibility criteria Psychocardiological subsample**

• The presence of a cardiac disease that requires rehabilitation by a specialist, e.g. specific cardiac arrhythmias, CHD, heart failure, at the same time, the presence of a mental disorder, diagnosed according to ICD-10 criteria by the clinician.

**Exclusion criteria**

• Aged under 18 years,
• insufficient knowledge of the German language to fill in the questionnaires
• patients with current psychotic symptoms, substance dependence or abuse, and organic brain disorders,
• patients in the acute phase of cardiac disease, e.g. follow-up treatment after heart transplantation or coronary surgery during the last 6 months.

### 2.2. Measures

#### 2.2.1. Beck depression inventory II (BDI-II), German version

The BDI-II (25, 26) is a well established self-report questionnaire for assessing the severity of depressive symptoms that is based on the diagnostic criteria of the DSM-IV. The BDI-II comprises 21 questions about symptoms in the last two weeks. The results represent the severity of the depressive symptoms. A total value of 13 or more is assessed as mild but clinically relevant symptomatology, values ranging from 20 to 28 are considererd as moderate and scores ranging from 29 to 63 as severe symptoms.

#### 2.2.2. The hamburg module for the assessment of psychosocial health in clinical practice (HEALTH-49)

The HEALTH-49 (27, 28) consists of 49 items and is a self-report questionnaire that provides a multidimensional assessment of various symptoms of mental illness and psychosocial functioning. The items assess depression, somatoform complaints, phobias, well-being, interaction difficulties, self-efficacy, activity and participation and social support and stress. The scales are partly based on varying response formats. In this study, we used only those HEALTH-49 subscales that either measure psychopathology or activity and participation in occupational/social life. Results of the HEALTH-49 scales social support and social stress are therefore not reported. The following cut-off scores are used: depression ≥0.66, somatoform complaints ≥0.987, phobia ≥0.255, interactional difficulty ≥1.0, well-being ≥1.821, Self-efficacy ≥1.364, Activity and participation ≥1.136.

#### 2.2.3. Work related/socio-medical parameters

The socio-medical parameters relevant at admission and discharge (Table 1) were routinely recorded as part of the rehabilitation. People who were able to work at least six hours a day were considered to be able to work full time. The performance assessment was carried out separately for the reference occupation (last job subject to social insurance contributions) and the general labour market. If the daily performance capacity is less than three hours per day, this is referred to as a reduced capacity, which often leads to a pension for reduced earning capacity, as long as there is no further capacity for the general labour market. The assessment of the ability to work at the time of discharge as well as the performance assessment was carried out by the rehabilitation team under the supervision of specialist physicians trained in social medicine.

**Table 1:** Descriptives.

Variables	Psychosomatic-subsample *n* = 49 (32.7%)	Post-COVID-subsample *n* = 51 (34.0%)	Psychocardiological-subsample *n* = 50 (33.3%)	*p*
*Demographic characteristics*				
Mean age (SD)	50.82 (9.37)	51.29 (9.89)	54.84 (7.70)	*
Gender (% female)	77.6%	76.5%	76.0%	-
Married	26.1%	12.1%	8.1%	*
Outpatient Psychotherapy before admission	89.8%	70.6%	80.0%	*
No education	2.1%	–	–	–
Lower vocational/general secondary education	2.1%	–	4.0%	–
Intermediate vocational education	70.8%	54.9%	70.0%	*
Higher education	25.0%	45.2%	28.0%	*
*Work characteristics*				
Mean weeks of sick leave before admission	28.6% < 3 months	41.0% < 3 months	42.0% < 3 months	–
	14.3% 3–6 months	13.7% 3–6 months	2.0% 3–6 months	
	51.0% > 6 months	43.1% > 6 months	56.0% > 6 months	
	6.1% not employable	2.0% not employable	0.0% not employable	
Unable to work on admission	65.3%	54.9%	62.0%	–
On the job	34.7%	45.1%	38.0%	–
*Return to work*				
Ability to work discharge	30.6% able to work	35.3% able to work	32.0% able to work	–
Gradual reintegration	10.2%	11.8%	8.0%	–
Total return to work after rehabilitation	40.6% return to work	47.1% return to work	40.0% return to work	*
*Socio-medical limitations*				
Suspended occupational performance last job	28.6%	9.8%	30.0%	*
Suspended occupational performance general labor market	20.4%	3.9%	16.0%	*

Significant group differences are indicated as follows: * < .00, ** < .000 – not sig.

#### 2.2.4. Six-minute walk test (6MWT)

The 6MWT is used to assess and control cardiovascular and pulmonary performance. In the test, the patient has to walk for 6 min over an incline-free circuit or a walkway of at least 30 m in length. The goal for the patient is to cover as much distance as possible in the given time (29). In addition, just before the start of the 6MWT and at the end of the 6 min, respiratory distress is determined using the Borg CR10 scale (30). The recorded distance can be used as a relevant global parameter for cardiopulmonary performance. A systematic review (31) of studies using 6MWT on healthy individuals shows that the average distance for women varies between 386 m (32) and 659 m (33). In healthy men, the average distance travelled ranges from 429 m (32) to 735 m (33). In a recent study of 6MWT in patients with severe lung disease (COPD) the average walking distance was 260 m (±107 m) and ranged from 64 m to 480 m (34).

## 3. Statistical analysis

Statistical analysis was conducted using SPSS 28 (35) for windows. Since patients were admitted to the departments of different sizes and included in the study at the same time, sub-samples were formed for each of the psychosomatic and cardiological patients by applying case-control-machining (FUZZY method) in order to obtain comparable samples to the post-COVID patients. Due to the significantly higher risk of developing post-COVID in women, case-control matching for gender was performed. Furthermore it was taken into account that only patients were included who were admitted at the same time. The sample characteristics and the description of the work-related parameters were determined using descriptive statistics (frequency analysis). Statistical analyses of the Likert-scaled items and scores included descriptive measures in terms of means (M) and measures of dispersion [standard deviation (SD)]. Explorative bivariate methods were applied when comparing e.g., different profession groups using the Mann–Whitney *U* test for independent ordinal data and metric data in the case of missing normal distribution. The general linear model (GLM), respectively MANOVAs were used for calculating differential outcomes of symptoms between the subggroups as well as within the subgroups from admission to discharge. Effect sizes (Cohen's d) were calculated based on estimated means and the pooled standard deviation from the observed means. The significance level was set to *α* = .05 on both sides and corrected according to Bonferroni to avoid α-error accumulation. Effect sizes of Cohen's *d* = .2 were considered small, .5 medium and .8 strong.

## 4. Results

### 4.1. Sample characteristics

During the period from September 2021 to March 2022, 458 patients were included (63.5% psychosomatic patients, 25.3% psychocardiological patients, 11.1% post-COVID patients). The gender and age distribution in the matched sample was typical for a rehabilitation population with a slight excess of female patients (60.3%) and an average age of 51.95 years (SD = 9.89). After applying case-control-matching for gender a sample of *n* = 150 patients was included (32.7% psychosomatics, 33.3% psychocardiology, 34.0% post-COVID). The gender and age distribution in the matched sample was typical for post-COVID with a clear majority of female patients (76.7%); this is in line with the results of other studies which find a higher risk of developing post-COVID-syndrome in women. In the post-COVID subgroup the average age was 52.32 years (SD = 9.16). The psychocardiological patients were slightly older than the psychosomatic and post-COVID patients. Post-COVID patients had a significantly higher proportion of higher education. This could be due to the fact that this patient group is more likely to suffer from cognitive impairments or to find their way to rehabilitation more quickly due to their knowledge of help options. All patients had an existing employment contract. With regard to the other socio medical data, there were no significant group-differences in term of sick leave and ability to work before start of rehabilitation. There were significant differences in work ability at discharge: post-COVID patients were discharged fit for work more often than patients in the other two subgroups. Post-COVID patients also differed significantly from the other two groups in the socio-medical performance assessment. Post-COVID patients were three times less likely than psychosomatic or psychocardiological patients to have their occupational performance suspended. The Participant's caracteristics are depicted in Table 1.

### 4.2. Symptom load on admission

In the BDI as a measurement of depressive symptoms, psychosomatic patients showed significantly increased values, while these were moderate in psychocardiological patients and rather mild -but still clinically relevant- in post-COVID patients.

In HEALTH-49, the post-COVID patients were again less burdened than the psychosomatic and psychocardiological patients. This was not the case concerning activty and participation, here post-COVID patientes were as burndened as the two other patient groups. Further results of the analysis can be found in Table 2.

**Table 2:** Symptomatology – differences between the groups and within the groups from admission to discharge.

Variables	*Between group differences*						Psychosomatic-subsample *n* = 49 (32.7%)				Post-COVID-subsample *n* = 51 (34.0%)				Psychocardiological-subsample *n* = 50 (33.3%)			
	Admisssion			Discharge			Admisssion	Discharge			Admisssion	Discharge			Admisssion	Discharge		
	P	C	K	P	C	K	M (SD)	M (SD)	*p*	*d*	M (SD)	M (SD)	*p*	*d*	M (SD)	M (SD)	*p*	*d*
*BDI-II*	C	P	–	–	–	–	28.93 (12.66)	19.83 (14.52)	**	.89	19.29 (9.03)	13.83 (8.95)	**	.81	24.47 (10.02)	18.18 (12.73)	**	.59
*HEALTH-49*																		
Somatoform Complaint	–	–	–				1.89 (.96)	1.35 (.96)	**	1.00	1.52 (.73)	1.18 (.82)	**	.60	1.82 (.90)	1.37 (.86)	**	.67
Depression	C	P	–	–	–	–	2.03 (.98)	1.28 (.95)	**	.97	1.06 (.83)	.65 (.67)	**	.60	1.78 (.104)	1.26 (1.15)	**	.68
Phobia	C	P	–	C	P	–	1.22 (1.10)	.86 (.98)	*	.43	.56 (.60)	.36 (.47)	*	.34	.89 (.92)	.68 (.87)	–	–
Well-being	–	–	–	–	–	–	1.75 (.87)	1.19 (.88)	**	.98	1.10 (.57)	.78 (.53)	**	1.22	1.55 (.77)	1.14 (.83)	**	.70
Interactional difficulties	C	P, K	C	C	P, K	C	2.81 (.77)	2.04 (.99)	**	.66	2.60 (.77)	1.70 (.67)	**	.81	2.82 (.70)	2.08 (.97)	**	.85
Self-efficacy	–	–	–	–	–	–	2.55 (.96)	1.87 (.97)	**	.65	1.33 (.83)	.83 (.81)	**	.69	1.87 (.99)	1.48 (1.08)	*	.44
Activity & participation	–	–	–	–	–	–	2.28 (.87)	1.80 (.98)	**	.83	2.20 (.93)	1.62 (.86)	**	1.06	2.61 (.79)	1.97 (1.11)	**	.79
*Somatic parameter*																		
6MWT						* *	–	–	–	–	421.92 (98.90)	498.35 (101.14)	**	1.22	–	–	–	–

Significant group differences are indicated as follows: * < .00, ** < .000 – not sig.; Cohen's d effect sizes:.2 small,.5 medium,.8 large; P, psychosomatic patientes; C, post-COVID patients, K, psychocardiological patients.

### 4.3. Course of symptoms

#### 4.3.1. Symptomatology form t1 to t2 - differences within the groups

In all patient groups, the severity of symptoms was significantly reduced from admission to discharge in nearly all complaint domains. The psychosomatic and psychocardiological patients, as well as the patients with post-COVID-syndrome were able to benefit from the rehabilitation. However, differences were found in the symptom severity of the respective groups. Here, psychosomatic patients and psychocardiological patients were most severely stressed at the beginning of rehabilitation. At discharge, all three patientgroups were comparably burdened, exept for HEALTH-49 phobia, interactionel difficulties and social stress. In these parameters post-COVID patients were significant less burdened than psychosomatic patients. In post-COVID patients and psychosomatic patients the rehabilitation had large effects on depressive symptoms, whereby the effects in psychocardiological patients were only medium. The rehabiliation had overall medium to large effects in all HEALTH-49 domains exept for self-efficacy in psychocardiological patients. There was also a strong positive effect on activity and participation in post-COVID patients.

For a closer look at HEALTH-49 activity and participation at the individual case level, a reliable change index of.67 was used (Rabung et al., 2016). The following results were found: Post-COVID patients benefited from rehabilitation program in activity and participation to a relevant extent in 56.52% and deteriorated in 0.00%. Psychosomatic and psychocardiological patients could only achieve improvements in activity and participation in 42.85% and 40.00% respectively, and even deteriorated by 9.52% (psychosomatic) and 11.11%. The percentage of patients who neither improved nor worsened was approximately the same in the psychosomatic and the psychocardiological subgroup. For the exact results, see Table 3 in the appendix.

**Table 3:** Individual values in HEALTH-49 activity & participation.

Psychosomatic subsample (*n* = 42)			Post-COVID subsample (*n* = 46)			Psychocardiological subsample (*n* = 45)		
Admission	Discharge	RCI	Admission	Discharge	RCI	Admission	Discharge	RCI
3.83	1.83	2.00^a^	2.33	2.33	.00	.67	.00	.67^a^
3.17	1.83	1.34^a^	3.83	3.17	.66	2.17	2.33	.16
3.00	1.17	1,17^a^	1.00	.17	.83^a^	1.83	1.00	.83^a^
3.50	1.00	2.50^a^	3.33	2.17	1.16^a^	2.67	2.00	.67^a^
2.50	3.17	.67^a^	1.33	1.00	.33	.67	1.00	.27
3.00	1.17	1.83^a^	2.17	.67	1.50^a^	2.50	1.00	1.50^a^
2.17	.67	1.50^a^	1.67	.00	1.67^a^	3.00	1.83	1.17^a^
.33	.33	0.00	1.83	1.50	.33	2.17	1.33	.84^a^
1.83	1.67	.16	2.00	.33	1.67^a^	3.83	3.17	.66
2.33	2.33	.00	1.67	2.33	.66	3.83	3.17	.66
3.00	2.00	1.00^a^	3.33	2.50	.83^a^	1.50	.83	.67^a^
2.50	1.17	1.33^a^	2.83	1.83	1.00^a^	2.00	3.00	1.00^a^
3.17	2.00	1.17^a^	2.67	.67	2.00^a^	1.17	.00	1.17^a^
.83	.00	.83^a^	3.33	2.33	1.00^a^	2.83	1.00	1.83^a^
2.00	2.83	.83^a^	2.83	1.00	1.83^a^	1.83	2.83	1.00^a^
3.33	2.83	.50	2.00	1.00	1.00^a^	1.67	2.17	.50
1.17	.33	.84^a^	1.33	1.00	.33	1.83	1.50	.33
2.33	2.17	.16	2.33	2.83	.50	2.83	2.50	.33
1.50	1.33	.17	2.83	2.33	.50	.83	1.50	.67^a^
1.33	1.67	.34	1.17	1.17	.00	.83	1.50	.67^a^
.83	1.00	.17	2.50	1.83	.67^a^	2.50	2.50	.00
3.33	3.50	.17	3.50	2.83	.67^a^	2.50	2.50	.00
.50	1.00	.50	2.67	.83	1.84^a^	1.33	.17	1.16^a^
3.17	.83	2.34^a^	2.67	2.00	.67^a^	2.17	1.33	.84^a^
2.00	2.33	.33	2.33	1.33	1.00^a^	2.67	2.83	.16
2.00	1.33	.67^a^	1.83	1.33	.50	2.67	2.83	.16
3.00	2.67	.33	2.50	1.17	1.33^a^	2.67	2.50	.17
1.83	3.00	.17	2.83	1.83	1.00^a^	1.67	3.17	1.50
2.00	.67	.33	1.83	.17	1.66^a^	1.67	3.17	1.50
1.17	.33	.84^a^	2.67	3.00	.33	2.83	2.50	.33
2.17	.67	1.50^a^	1.67	1.50	.17	3.33	.67	2.66^a^
3.67	3.67	.00	2.00	2.17	.17	1.50	2.00	.50
3.17	2.50	.67^a^	1.83	1.33	.50	3.17	3.00	.17
1.67	2.33	.66	3.17	1.67	1.50^a^	3.17	3.00	.17
3.00	3.67	.67^a^	2.33	1.00	1.33^a^	1.83	2.33	.50
1.67	2.50	.83^a^	2.17	.17	2.00^a^	2.33	.83	1.50^a^
3.00	3.17	.17	1.83	1.00	.83^a^	2.33	2.00	.33
2.00	2.33	.33	3.00	2.00	1.00^a^	2.33	2.00	.33
2.17	.83	1.34^a^	2.00	2.17	.17	2.50	2.00	.50
2.00	1.83	.17	1.83	1.67	.16	2.50	.50	2.00^a^
2.33	1.67	.66	1.83	2.17	.34	3.00	3.67	.67^a^
2.67	2.50	.17	2.00	2.17	.17	2.83	1.00	1.83^a^
			2.00	1.50	.50	2.33	.00	2.33^a^
			2.50	1.67	.83^a^	2.50	1.17	1.33^a^
			2.33	1.33	1.00^a^	2.50	1.17	1.33^a^
relevant worsening: 9.52%			relevant worsening: 0.00%			relevant worsening: 11.11%		
constant: 47.61%			constant: 43.48%			constant: 48.89%		
relevant improvement: 42.85%			Relevant improvement:56.52%			relevant improvement: 40.00%		

A reliable change index (RCI) ≥ .67 indicates a relevant change.

^a^ Relevant change.

**Figure 1:**
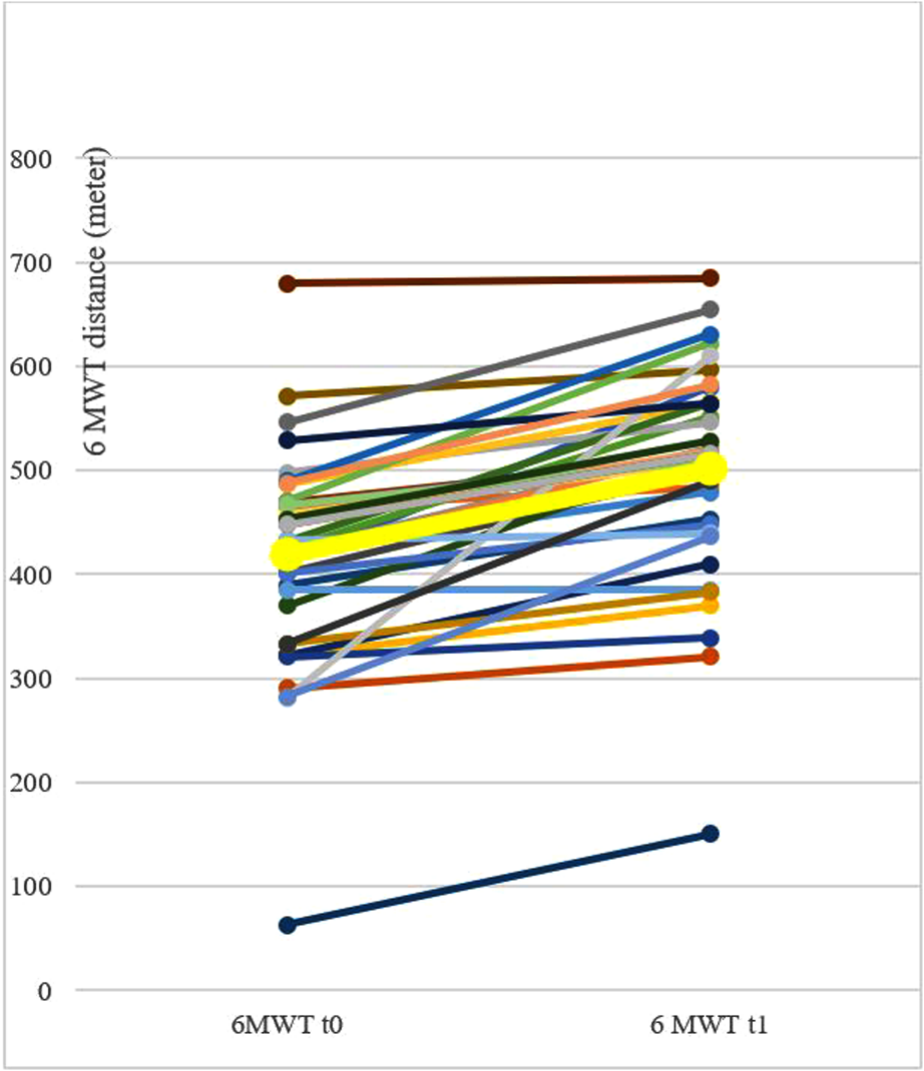
Development of 6MWT during rehabilitation of post-COVID patients. Development of 6 Min Walk Test (6MWT) of the single patients during rehabilitation of post-COVID (colored thin lines, *n* = 39) in the beginning (t0) and in the end of the rehabilitation (t1) and mean value (thick yellow line).

In the 6MWT out of 51 post-COVID patients, comparable data could be used in 39 cases (76%). The patients could cover significantly more distance (large effect). There was not a single patient who deteriorated in terms of their walking distance (6MWT) during moderate physical training. Figure 1 shows the improvements in the 6MWT. The individual walking distances of the participating post-COVID patients can be found in Table 4 in the appendix. The results of the symptom course from admission to discharge are depicted in Table 2.

**Table 4:** Individual results of the post-COVID patients in the 6MWT.

Pat	6MWT 1	6MWT 2	Dif.	Pat	6MWT 1	6MWT 2	Dif.	Pat	6MWT 1	6MWT 2	Dif.
1	412 m	580 m	+168 m	14	419 m	520 m	+101 m	27	282 m	609 m	+327 m
2	465 m	484 m	+19 m	15	498 m	546 m	+48 m	28	457 m	508 m	+51 m
3	421 m	521 m	+100 m	16	487 m	565 m	+78 m	29	433 m	439 m	+6 m
4	322 m	370 m	+48 m	17	385 m	385 m	+0 m	30	467 m	510 m	+43 m
5	420 m	479 m	+59 m	18	470 m	622 m	+152 m	31	529 m	564 m	+35 m
6	425 m	551 m	+126 m	19	320 m	339 m	+19 m	32	680 m	685 m	+5 m
7	322 m	410 m	+88 m	20	290 m	320 m	+30 m	33	333 m	490 m	+157 m
8	469 m	512 m	+43 m	21	546 m	654 m	+108 m	34	448 m	516 m	+68 m
9	402 m	500 m	+98 m	22	333 m	383 m	+ 50 m	35	64 m	150 m	+86 m
10	572 m	597 m	+25 m	23	490 m	631 m	+141 m	36	453 m	528 m	+75 m
11	390 m	453 m	+63 m	24	432 m	564 m	+132 m	37	283 m	436 m	+153 m
12	370 m	516 m	+146 m	25	467 m	512 m	+45 m	38	488 m	582 m	+94 m
13	401 m	448 m	+47 m	26	453 m	518 m	+25 m	39	448 m	516 m	+68 m

Pat, patient, Dif, Difference, 6MWT, six-minute-walking test.

#### 4.3.2. Symptomatology – differences between the groups post-COVID, psychosomatic, psychocardiology

Between the groups at admission, significant more depressive symptoms were found in psychosomatic patients; patients with post-COVID syndrome and psychocardiological patients did not differ from each other and were moderately burdened. At discharge, this difference was still present between post-COVID and psychosomatic patients. Subgroup differences between psychosomatic and psychocardiology patients and the patient group post-COVID-syndrome were also present in the different domains of the HEALTH-49. These group differences dissolved at discharge between post-COVID and psychocardiology patients in all symptom areas except for psychosomatic patients, who continued to be significantly more burdened in the domains Phobia and Interactional difficulties. The data show that patients with post-COVID syndrome are significantly less burdened in the psychopathological measures than psychosomatic patients, but have comparable limitations in the areas of activity and participation. The 6MWT was only conducted in the post-COVID group. A comparison with the other patient-groups was not possible. The results of the group differences can be seen in Table 2.

## 5. Summary and discussion

This study shows that patients with post-COVID-syndrome are in the clinically relevant range with regard to psychological symptoms, but are less burdened than psychosomatic and psychocardiological patients. However, the strain is comparably high on the ICF-oriented activity and participation scale, which is particularly relevant for rehabilitation. This suggests a multifactorial understanding of post-COVID symptoms, in which psychological complaints represent important subcomponents. Viewed across all parameters, the multimodal concept of rehabilitation was shown to be comparably effective in post-COVID patients as in psychosomatic and psychocardiological patients; the effect sizes were consistently in the medium to high range. Post-COVID patients benefited particularly in terms of activity and participation. There were no indications of post-exercise malaise in the course of rehabilitation.

Some studies on the rehabilitation of COVID-19 patients, mostly after severe lung involvement or in post-acute care, have been published (36–39). This study is, to our knowledge, the first to compare a special multidisciplinary rehabilitation concept with already well-established and evaluated rehabilitation concepts for psychosomatic disorders or disorders with a high somatic component and psychological co-involvement (13). Even though patients with post-COVID syndrome were less psychopathologically burdened, they showed comparable limitations in the socially and socio-medically relevant areas of activity and participation. At the same time, the significant improvement in depressive symptoms and self-efficacy as well as the considerable increase in well-being and especially in activity and participation are evidence of the positive effect of post-COVID rehabilitation. In conclusion it can be stated that patients with post-COVID syndrome benefit significantly from rehabilitation. For example the effect on depression was less pronounced in the psychocardiological subsample than in the psychosomatic subsample. These results also correspond to the current literature: the literature shows that both psychotherapy and antidepressants have different effects in patients with cardiac comorbidity than in those with solely mental illness. There seems to be no clear evidence of effect for psychotherapy (40). Data suggest that women with cardiological diseases and depressive symptoms have a stronger response to treatment than their male equivalents (41). In the case of coronary heart disease, effects of antidepressants could be demonstrated (42, 43), although antidepressant treatment seems to have less effect in heart failure patients than in patients with only mental illness (44, 45). There is also evidence that psychocardiological patients show deficits in emotion regulation, which in turn increases the risk of depression and a worse course of cardiac disease (46). With this in mind, our findings of a lower response of psychocardiological patients to rehabilitation do not seem surprising. Another explanation for the difference in treatment response could be that, for example, although psychocardiological patients show depressive symptoms, these could be attributed to the physical effects of cardiac disease (e.g., reduced drive in heart failure), effects of medication or random events (e.g., temporary mood lulls due to job loss) rather than to a substantial psychological disorder. It is therefore possible that there could be some overdiagnosis of mental disorders in cardiac patients due to misinterpretation of patients reported symptoms (24) and therefore said patient group is less responsive to psychotherapeutic interventions. Another result should be emphasised: in the area of activity and participation, 56.52% of the post-COVID patients benefited clinically relevant from rehabilitation, whereas only 42.85% of the psychosomatic patients and 40.00% of the psychocardiological patients were able to benefit from the rehabilitation program. Due to the approximate 50% of patients which did not benefit significantly during the five weeks of rehabilitation, one could have the critical idea “rehabilitation does not really work”. On the one side this finding could be explained by chronicity of the illness (especially with psychocardiological and psychosomatic patients), the long persisting character of the post-COVID symptomatology (47) and the short five weeks inpatient treatment. We would state that physical and mental capacity training may need more time for reaching observable effects as compared with short term effects from support of wellbeing. We state that, with treatments which focuse on strengthening capacities for the long-term perspective, effects like work participation improvement, cannot appear within all patients within the given short period of rehabilitation. On the other side on admission 34.7% of the psychosomatic patients, 38.0% of the psychocardiological patients and 45.1% of the post-COVID patients were able to work - on discharge in all patients groups were was an amelioration concerning workability. 40.6% of the psychosomatic patients, 40.0% of the psychocardiological patients and 47.1% of the post-COVID patients returned to work after rehabilitation. Therefore it can be said, that there are some changes, especially in psychosomatic patients. At the same time, it should be said that the changes achieved in rehabilitation are not sufficient and aftercare services especially for post-COVID patients are urgently needed.

When dealing with the rehabilitation of post-COVID patients, further rehabilitation indications and their effectiveness must be considered against the background of multi-systemic symptoms. Most patients report neurological and neurocognitive symptoms. There are some studies that have suggested strategies to reduce the neuropsychological impact of the symptoms linked to post-COVID syndrome(48, 49), which included in health resort medicine and may be suitable a to recover disabilities. These strategies may include therapeutic massage, water massage, physical modalities, numerous forms of exercise such as water exercise, breathing, balance, and muscle-strengthening exercises, health education, psychological interventions, and treatments complementary to balneotherapy (48). Concepts of motor rehabilitation expect promising results (50). Conceptually, cardiopulmonary rehabilitation offers individualized training individualised according to the F.I.T.T. principles (frequency, intensity, time, and type). The intensity individualized and well monitored includes Aerobic exercise on a cyclo-ergometer, strengthening exercises with weight machines, free weights and/or elastic bands and at the end of the monitored training session respiratory exercises including pursed-lip abdominal breathing exercise and inspiratory muscle training (51). Another study shows that aquatic exercises technique seemed to contribute to recovery of impaired upright posture and motor function, normalizing the walking pattern (52).

Against the background of the discussion about possible damage due to post-exercise malaise, the result in the 6MWT should be emphasised, which shows an improvement in physical resilience under a moderate physical training. The walking distances of post-COVID patients in the 6MWT can be considered just about average compared to values of healthy persons; compared to COPD-patients, the average values of post-COVID patients appear good. The significant increase in walking distance as part of the rehabilitation measure and above all the observation that not a single patient deteriorated, also indicates that there is no need for fearing post-exercise malaise in context of a well dosed and monitored moderate physical strain. It can therefore be argued that although post-COVID rehabilitation is not a causal treatment, it does address the key issues to help patients with post-COVID-syndrome return to participation in society.

## 6. Strength and limitations

The strengths of the study include the opportunity to compare post-COVID patients with other rehabilitants in terms of symptom burden in the cause of rehabilitation.

Also, limitations of this study should be addressed. Firstly, the present study is not a randomised controlled design, but a retrospective data evaluation. Thus, causal statements on the actual effectiveness of rehabilitation are not possible. Our data can only provide indications for later RCTs. Nevertheless, our results are of scientific value, especially since there is no reliable data available in this area so far. Second, there is a relatively small sample sizes with 51 post-COVID patients, compared to 50 psychocardiological patients and 49 psychosomatic patients. This can be explained by the recently established treatment programm of post-COVID rehabilitation, which was integrated into the multidisciplinary treatment concept of psychocardiology and therefore only provides a total of 12 treatment units. In the present study, there is also no control group without intervention, so that time effects, in the sense of a natural regeneration and reduction of symptoms, cannot be examined. At the same time, ethical and legal reasons prohibit the withholding of a rehabilitative treatment; moreover, there is a right to rehabilitation in Germany. Since the data collection took place within the framework of routine diagnostics of the clinic routine, a waiting list control group was also not feasible. In this study, neurocognitive disorders were not yet systematically recorded; a systematic recording is planned in a follow-up study on post-COVID rehabilitation (53).

## 7. Conclusion

The results of this study show that rehabilitation patients with post-COVID-syndrome have a significantly elevated, but lower psychological burden than patients in psychosomatic or psychocardiological rehabilitation. Post-COVID appears to be a mutlifactorial clinical picture that includes somatic and psychological components and has socio-medical and social implications (8, 54).

It was also shown that although rehabilitation is not a curative procedure, patients with post-COVID-syndrome improve significantly during the stay in rehabilitation. Although post-COVID patients are significantly less burdened in psychopathological symptom areas the high burden in activity and participation is striking. In addition to the general symptom improvement, the clear benefit of post-COVID patients in the socio-medically relevant areas of activity and participation should also be emphasised. To sum up, it can be said that:

1. Post-COVID patients in inpatient rehabilitation show a lower psychological burden than psychosomatic or psychocardiological patients, but are also clinically significant burdened at the start of rehabilitation.
2. Despite the lower psychological burden, patients with post-COVID syndrome are similarly limited in terms of activity and participation as well as in terms of social medicine as psychosomatic or psychocardiological patients.
3. Post-COVID patients improve strongly in terms of activity and participation, moderately to strongly in psychological complaints and also strongly in the somatic parameter of the 6MWT.
4. Despite rehabilitation success with symptom reduction and increase in physical performance, about 50% of post-COVID patients were discharged unable to work, mainly due to persistent cognitive deficits.
5. There was no evidence of post-exercise malaise or longer-term deterioration in health status, neither in the clinical course nor in the 6MWT or in activity and participation.

In summary of the results it can be assumed that multimodal rehabilitation is not only helpful, but also safe for post-COVID patients.

Although there has been some research on interventions for patients suffering from post-COVID-syndrome, there is still a lack of knowledge about how to treat effectively. In addition, the lack of studies with good methodological quality focusing on interventions especially for post-COVID-syndrome calls for further research. There is a strong need to support this population adequately, due to their high levels of mental distress and risk for developing anxiety, depression symptoms or an exercise phobia and chronic stress intolerance.

## Data Availability

The data analyzed in this study is subject to the following licenses/restrictions: Since the data that will be used in the targeted study in the future will be data on insured persons of the German Pension Insurance and strict data protection exists, these data are not publicly accessible, but can be made available by the corresponding author in a form adjusted for personal data upon justified request. Requests to access these datasets should be directed to alexa.kupferschmitt@charite.de.
